# Micro-Organisms as a Cause of Chronic Disease

**Published:** 1900-02-17

**Authors:** 


### Editor's Letter-Box.

[Our Correspondents are reminded that prolixity is a great bar to publication, and that brevity of style and conciseness of statement greatly facilitate early insertion.]

### MICRO-ORGANISMS AS A CAUSE OF CHRONIC DISEASE.

Dr. T. H. Godfrey writes: The article under this heading in your issue of February 3rd interested me very much, as for years past I have speculated on this subject. But in the case of cirrhosis of liver must we not admit some closer causative relation to it of alcoholic excess than the production of a gastric and intestinal catarrh, which invites or allows germ poisoning, which then, you suggest, causes cirrhosis? Otherwise, how can we explain the great frequency of chronic alimentary catarrh in the non-alcoholic which, at all events in most such cases, does not lead to cirrhosis? That they also, through their catarrh, suffer from an abnormal amount of germ-poisoning I believe to be a fact. But why do they not more commonly get cirrhosis of liver? Must we not conclude that cirrhosis is either the direct effect of alcohol on the liver structures or that the action of alcohol on the lymphatic "defensive" structures of our alimentary organs is such as to frequently render them unable to cope with some particular germ, which in its turn causes the cirrhosis? Of course, this is mere hypothesis, but would explain how some inveterate topers escape cirrhosis, why temperate people occasionally get it, as, for instance, the recorded case of the imbecile who had never tasted alcohol, and why the non-alcoholic who suffer from chronic alimentary catarrh are not specially liable to cirrhosis. In spite of this criticism, I agree with you that many chronic diseased conditions are but expressions of a past contest with an infection which did not gain a sufficient footing to definitely prove its presence or nature. Every general practitioner must notice how frequently in times of epidemics those in special risk of infection get slight ailments, but not the definite disease. The germs gain admission, but are destroyed by the defensive lymphatic tissues at slight present cost, yet may insidiously leave a local disability productive, possibly, of future chronic trouble. More cheering is the thought that this defeated infection probably has its influence in protecting the individual from future attacks of that disease. If it is true, as stated, that medical men comparatively rarely suffer from infectious diseases, is it because their defensive cells are trained to destroy through being offered repeated small doses of germs? Another interesting question is whether the subjects of a defeated infection are at any time infectious to others. Presumably they are so, and this is a point which must be considered when anticipating how much benefit the country will derive from present methods of compulsory isolation and disinfection.

